# Encapsulation of Hydrogen Peroxide in PVA/PVP Hydrogels for Medical Applications

**DOI:** 10.3390/gels11010031

**Published:** 2025-01-02

**Authors:** Natalie Mounayer, Sivan Shoshani, Elena Afrimzon, Taly Iline-Vul, Moris Topaz, Ehud Banin, Shlomo Margel

**Affiliations:** Institute of Nanotechnology and Advanced Materials, Department of Chemistry, Bar-Ilan University, Ramat-Gan 5290002, Israel; natali_monayer@hotmail.com (N.M.); sivan.shoshani@biu.ac.il (S.S.); elena.afrimzon@biu.ac.il (E.A.); iv.taly@gmail.com (T.I.-V.); mtopazmd@yahoo.com (M.T.); ehud.banin@biu.ac.il (E.B.)

**Keywords:** PVA, PVP, hydrogel, controlled release, H_2_O_2_, medical applications, wound healing, polymers, biodegradable

## Abstract

Researchers have been investigating the physical and morphological properties of biodegradable polymer and copolymer films, blending them with other chemicals to solve challenges in medical, industrial, and eco-environmental fields. The present study introduces a novel, straightforward method for preparing biodegradable hydrogels based on polyvinyl alcohol (PVA) and polyvinyl pyrrolidone (PVP) for medical applications. The resulting PVA/PVP-based hydrogel uniquely combines the water absorbency, biocompatibility, and biodegradability of the polymer composite. For hygiene products and medical uses, such as wound healing, hydrogen peroxide (HP) was encapsulated in the PVA/PVP hydrogels for controlled release application. Incorporating PVP into PVA significantly enhances the hydrogel water absorbency and improves the mechanical properties. However, to mitigate the disadvantage of high water absorbency which could result in undesired early dissolution, efforts were made to increase the water resistance and the mechanical characteristics of these hydrogels using freeze–thaw (F/T) cycles and chemical crosslinking PVA chains with trisodium trimetaphosphate (STMP). The resulting hydrogels serve as environmentally friendly bio-based polymer blends, broadening their applications in medical and industrial products. The structural and morphological properties of the hydrogel were characterized using Fourier transform infrared spectroscopy (FTIR), environmental scanning electron microscope analysis (E-SEM), and water-swelling tests. The HP controlled release rate was evaluated through kinetic release experiments using the ex vivo skin model. The antibacterial activity of the hydrogel films was examined on four medically relevant bacteria: *Staphylococcus aureus*, *Enterococcus faecalis*, *Escherichia coli*, and *Pseudomonas aeruginosa*, with an adapted disk diffusion assay. Using this assay, we also evaluated the antibacterial effect of the hydrogel films over the course of days, demonstrating the HP controlled release from these hydrogels. These findings support further in vivo investigation into controlled HP release systems for improved wound-healing outcomes.

## 1. Introduction

Hydrogels are three-dimensional polymeric networks that contain at least 10% of aqueous solution, maintaining equilibrium through physical and chemical crosslinking within the hydrogel network. These structures exhibit high water absorption capacity, including superabsorbent types capable of absorbing water over 100% of their dry weight [1]. Hydrogels can occur naturally as polymer networks in materials such as collagen or gelatin, or they can be synthesized artificially [2]. Their attractive physicochemical properties, including mechanical resistance, swelling behavior, and moisture-holding capacity, make them suitable for a wide range of industrial applications, such as cosmetics, wastewater treatment, tissue engineering, drug release, biosensing, agriculture, biomedicine, food, and packaging [3,4,5].

Hydrogels exhibit remarkable physicochemical changes with minor alterations in the surrounding medium, such as chemical (pH, glucose, oxidants), biochemical (antigen, enzyme, ligand), and physical (temperature, pressure, light) diversions. These responses depend on the nature of the monomer, charge density, pendant chains, and degree of crosslinking [6]. It is important to note that water is a major component of hydrogels, and using low-concentration blends reduces the overall production cost, resulting in cost-effective and eco-environment-friendly materials that hold great potential in industrial applications [4,7,8,9,10,11].

Among various polymers, polyvinyl alcohol (PVA) and polyvinyl pyrrolidone (PVP) have been gaining increasing interest over the last century [6,8]. Their combination is acknowledged for its unique properties across a wide spectrum of biomedical applications, including drug delivery devices, implants, and wound dressings [8,9].

Polyvinyl alcohol (PVA) emerges as a semi-crystalline, hydrophilic, low-cost, non-toxic, and water-soluble polymer, that is biodegradable by microorganisms in both aerobic and anaerobic conditions [12]. Its remarkable film-forming, emulsifying, and adhesive properties, along with high tensile strength and flexibility, make it suitable for various applications in agriculture, biomedical, and pharmaceutical fields [13,14,15,16,17]. Moreover, PVA’s attributes include non-carcinogenicity, bio-adhesiveness, ease of processing, and recognition as a food additive [18,19,20].

On the other hand, polyvinyl pyrrolidone (PVP) is a hydrophilic synthetic polymer that is water-soluble and exhibits excellent physiological compatibility, enhancing its versatility for a multitude of applications. These include use as a plasma expander, binder in tablets, wetting agent, contact lenses, emulsifier, and in membranes, highlighting its potential in a biomedical context. PVP has been utilized in various domains, including the cosmetic, pharmaceutical, and food industries [21,22]. Despite its initial poor mechanical properties, PVP’s water solubility and capabilities enable its integration with other materials, resulting in improved mechanical properties [23,24,25].

**Figure 1:**
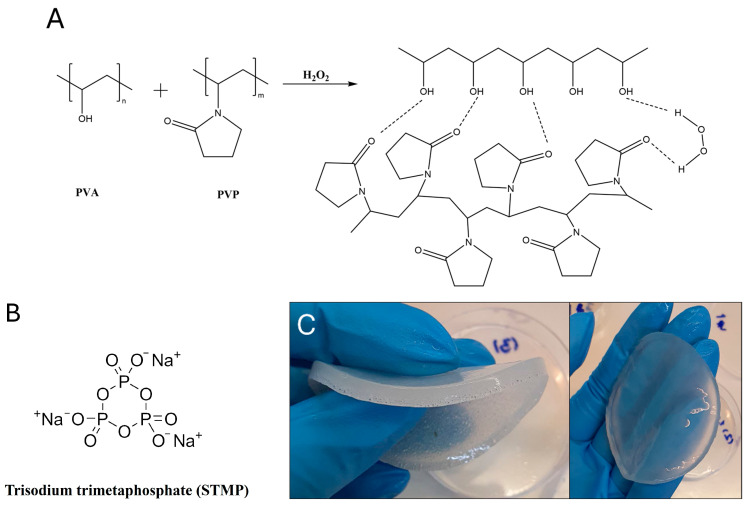
Hydrogen bond forming in PVA/PVP/HP hydrogels (**A**), chemical structure of STMP (**B**) and hydrogel molding (**C**).

PVA and PVP engage in intermolecular hydrogen bonding, specifically between the hydroxyl group of PVA and the carbonyl group of PVP [8,26] (Figure 1). The formed hydrogen bonds enhance mechanical properties [27] and form high-strength, physically crosslinked structures that can be molded into different stable shapes as shown in Figure 1C [7]. The mechanical properties of these hydrogels can be further improved by chemically crosslinking the polymer chains through chemical agents such as trisodium trimetaphosphate (STMP) [28] (Figure 1B) and by using freeze–thaw (F/T) cycles to physically strengthen the hydrogen bonds [29,30]. This results in a homogeneous structure with a flexible, porous network in the hydrogel, enabling the entrapment of functional substances soluble in aqueous solutions and solvents, such as drugs and active substances such as hydrogen peroxide (HP) [31,32]. This absorb-and-release behavior of hydrogels demonstrates their potential for controlled substance delivery [33,34].

The search for effective wound-healing alternatives is driven by critical concerns from both patient care and healthcare system perspectives [35]. Traditional wound care methods often fall short, especially when dealing with infected chronic or complex wounds, leading to prolonged healing times, frequent dressing changes, and increased infection risks [35]. These challenges result in significant physical discomfort, extended pain, and a diminished quality of life for patients, particularly those with conditions like diabetic ulcers or pressure sores. Consequently, there is an urgent need for innovative disinfection solutions that can accelerate wound healing and recovery, reduce pain, and lower infection risks, ultimately improving patient outcomes and quality of life. From an institutional perspective, managing infected chronic or complex wounds imposes a substantial burden on healthcare resources [36]. Traditional wound care often requires extended treatment periods and increased use of antibiotics, driving up costs associated with materials, medications, and healthcare personnel. The rise in antibiotic-resistant infections has compounded these challenges, making traditional methods less effective and more costly [37].

Therefore, hydrogels infused with antimicrobial agents offer a promising solution to infection risk in wound care [38]. Their ability to maintain a moist environment benefits cell proliferation and tissue regeneration, while presenting a risk for bacterial growth [39]. Incorporating antimicrobial agents, such as silver nanoparticles or broad-spectrum antimicrobial peptides [40], into hydrogels effectively combats common wound pathogens, such as *Enterococcus faecalis*, *Staphylococcus aureus*, *Escherichia coli*, and *Pseudomonas aeruginosa* [41]. This approach ensures sustained antimicrobial release, minimizing the need for frequent dressing changes.

Hydrogen peroxide (H_2_O_2_, HP) is widely used due to its low cost, oxidant effects, and the generation of reactive oxygen species (ROS) [42,43,44]. It demonstrates efficacy against a broad spectrum of microorganisms including bacteria, fungi and their spores state, viruses, protozoa, mycobacteria, nematode worms, prions, toxins, etc., and reacts rapidly in the presence of both organic and inorganic matter [45]. Notably, HP is environmentally friendly, as it decomposes into oxygen and water, leaving no toxic residues [46].

At concentrations ranging from 1% to 5%, HP acts as an antiseptic, aids in wound debridement, and promotes the healing process [47,48]. Concentrations of 1.5% to 3% HP are often required to effectively treat resistant infections, including multidrug-resistant (MDR) bacteria, biofilms, and high bacterial loads [49,50]. In other scenarios, lower concentrations may suffice. Infections can disrupt the wound-healing process at any stage, particularly during the inflammatory phase, leading to delayed healing and closure. Infection remains one of the primary obstacles to wound healing, clinically justifying the use of HP within a concentration range of 1.5% to 3%, despite its potential to induce oxidative stress and cytotoxic effects, which may impair wound healing [51]. Further clinical research is necessary to determine the optimal HP concentrations tailored to specific clinical infection scenarios.

PVA/PVP hydrogels are an ideal stabilizing carrier and release agent for HP, as hydrogen bonds are formed with PVA, while PVP forms a complex with HP [52,53]. Such encapsulation approaches have been suggested to mitigate the cytotoxic effect associated with higher HP concentrations [51]. Therefore, this study presents a novel application of PVA/PVP hydrogels encapsulated with HP as a controlled release device for wound healing purposes against pathogenic bacteria. Given the increasing interest in hydrogels [54,55], particularly PVA/PVP-based hydrogels, this method offers an innovative, straightforward, and cost-effective approach to facilitate and expand the practical application of PVA/PVP hydrogels in wound healing. This innovative approach offers a valuable addition to existing antimicrobial hydrogel technologies [56,57,58], without the requirement for additional external triggers for activation. These PVA/PVP hydrogels provide an immediate, sustained release of HP, enabling continuous antibacterial activity, where consistent antimicrobial action is needed. By allowing control over the HP release rate through crosslinking techniques, this study provides an adaptable solution that complements other approaches by reducing HP cytotoxicity while maintaining antimicrobial efficacy, enhancing the range of available options for medical industry applications.

Moreover, the effects of the physical and chemical changes in the hydrogel structure on the controlled release behavior of the HP and the effectiveness of the controlled release against typical pathogenic bacteria found in wound infections were examined [59,60,61,62]: *E. faecalis*, *S. aureus*, *E. coli,* and *P. aeruginosa*. The antibacterial activity of the hydrogels was examined using an adapted Kirby–Bauer disk diffusion assay, which is a widely used method in clinical microbiology laboratories for routine antimicrobial testing [63,64,65,66]. The alterations in controlled release behavior observed using the skin model in this study were influenced by both the degree and form of crosslinking. Physical crosslinking was affected by the number of freeze–thaw cycles, while chemical crosslinking was impacted by the concentration of the crosslinking agent, STMP, within the hydrogel composition. The skin model serves as an efficient, practical, and cost-effective alternative for investigating wound healing, particularly for burn wound applications [67]. It shares numerous characteristics with mammalian skin, and despite some differences in structural and physiological details, the fundamental cellular mechanisms and responses to chemical stimuli in human wounds are quite similar [67]. This makes the skin model a valuable tool for studying the healing processes associated with burn wounds. The hydrogels were characterized using Fourier-transform infrared spectroscopy (FTIR), scanning electron microscopy (E-SEM), and water-swelling behavior.

## 2. Results and Discussion

### 2.1. Preparation of the Different PVA/PVP/HP Hydrogels

Hydrogels were prepared using a consistent overall polymeric content of 21% (*w/v*), based on a previous study performed in our lab [34,68], and loaded with varying concentrations of HP (1.5%, 3%, and 5%). Some of these hydrogels were also chemically and physically crosslinked. Chemically crosslinked hydrogels were prepared with 10 and 20% (*w/v*%) crosslinking ratios of STMP to PVA. Physically crosslinked hydrogels were prepared through five cycles of freeze–thaw (F/T). These modified hydrogels were compared to the originally prepared hydrogels, which remained untreated. These modifications were performed to examine the effect of the physical and chemical changes in the hydrogel structure on the controlled release behavior of encapsulated HP and on physical properties.

**Figure 2:**
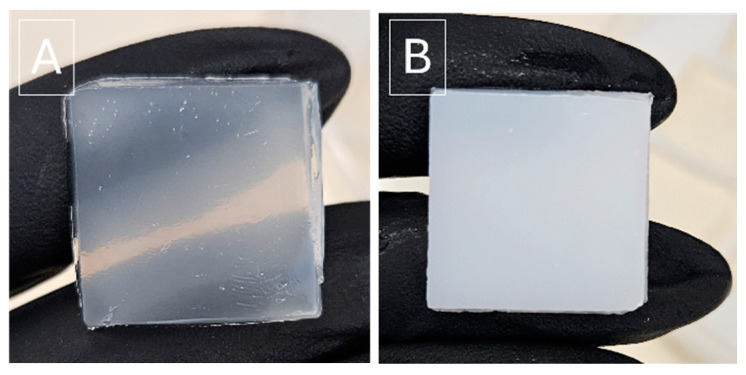
Digital images of PVA/PVP/HP-3 (**A**) and PVA/PVP/HP-3, 10% STMP (**B**).

Figure 2 demonstrates the effect of the concentration of the crosslinking agent, STMP, on the transparency of the PVA/PVP hydrogel. As the crosslinking agent concentration increases, the transparency of the films decreases. However, a higher concentration of the crosslinking agent STMP resulted in firmer and more elastic films, making them more durable and comfortable for use.

### 2.2. In Vitro: Agar Disk Diffusion Test to Assess the Antibacterial Activity of the Hydrogels

**Figure 3:**
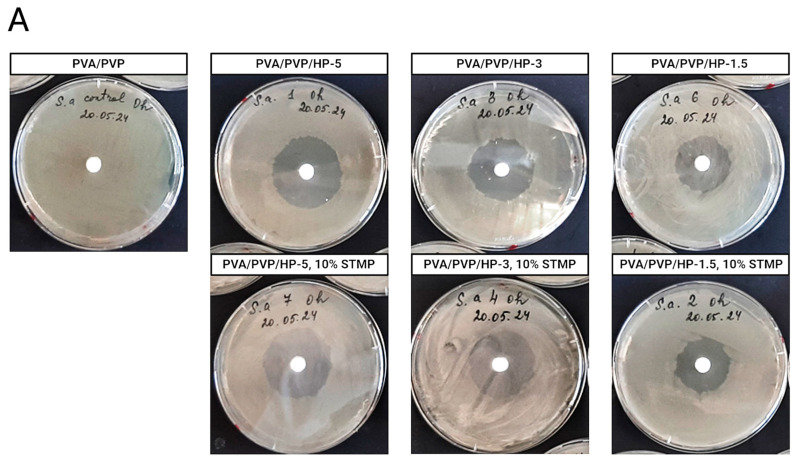

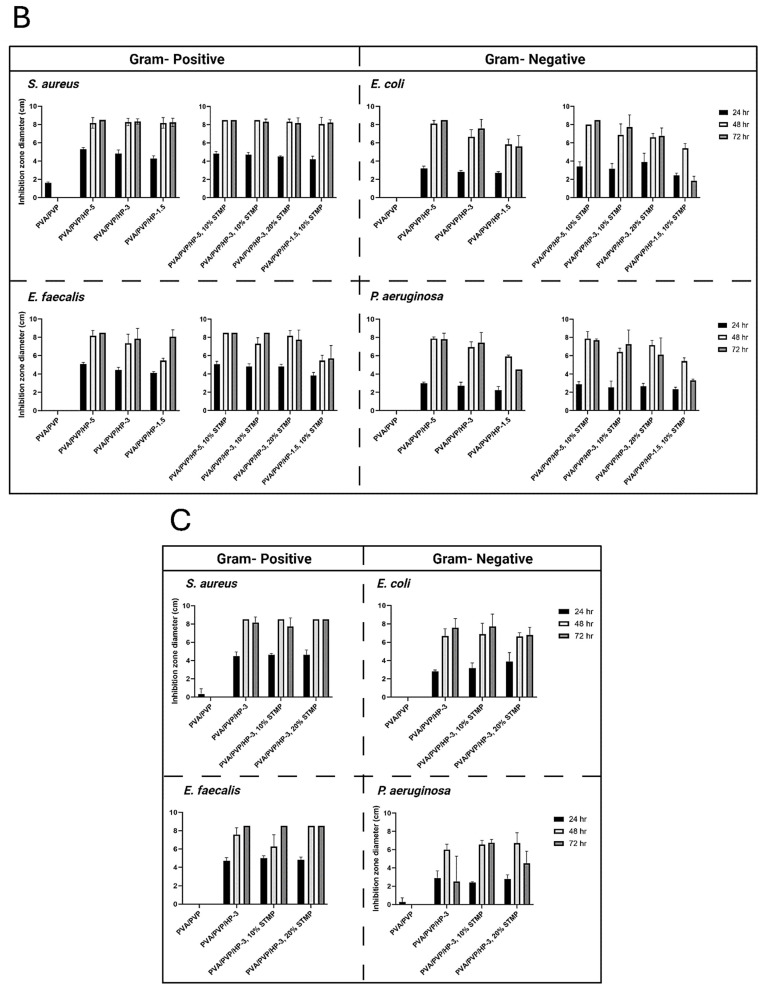
Representative image of the agar disk diffusion assay of the crosslinked and non-crosslinked hydrogels (**A**) and antibacterial effect represented by inhibition zone diameter over three days of 1 F/T cycle (**B**) and 5 F/T cycles hydrogels (**C**).

To evaluate the antibacterial properties of the hydrogels, we modified the disk diffusion assay to suit our experiment. Each type of hydrogel was placed separately at the center of three LB-agar Petri plates, allowing the material to diffuse through the agar over time. The antibacterial effect was monitored over a period of three consecutive days. Each day, starting from day 1, we plated the tested bacteria onto one of the plates, followed by incubation. The next day, an inhibition zone became visible (Figure 3A), indicating the antibacterial activity of the hydrogel from the previous day. This procedure was repeated with the second and third plates, which had been incubated for 48 and 72 h with the hydrogel, allowing for varying durations of diffusion. Accordingly, the inhibition zones received on the second and third plates corresponded to the antibacterial activity of the hydrogel on days 2 and 3, respectively.

Figure 3B presents the antibacterial activity of different hydrogels, PVA/PVP/HP-1.5, PVA/PVP/HP-3, PVA/PVP/HP-5, and their crosslinked variants, against four bacterial strains: *S. aureus*, *E. faecalis*, *E. coli*, and *P. aeruginosa*, with PVA/PVP serving as the control. In all cases, antibacterial activity was observed within the first 24 h. As expected, hydrogels with higher HP content exhibited larger inhibition zones, indicating greater antibacterial activity. This trend was also evident in crosslinked hydrogels prepared with 10% STMP. Notably, PVA/PVP/HP-3, 20% STMP displayed similar activity to PVA/PVP/HP-3, 10%, suggesting that a higher degree of crosslinking did not result in a more significant antibacterial effect.

Interestingly, on day 2, after 48 h of active material release, the antibacterial effect was more pronounced than after the initial 24 h. For most of the hydrogels, a direct correlation between HP content and inhibition zone diameter was observed on days 2 and 3. However, this pattern was not seen with *S. aureus* due to the physical size limitations of the plate.

Comparing the antibacterial effects on day 3 with day 2, a similar or greater effect was observed for most cases. However, for *E. coli* and *P. aeruginosa*, the lower HP-containing hydrogels (PVA/PVP/HP-1.5 and PVA/PVP/HP-1.5, 10% STMP) showed reduced antibacterial effects on day 3 compared to day 2. This might be due to the overall lower susceptibility of gram-negative bacteria to the hydrogels, which may be more prominent with lower active material present.

As mentioned, our findings indicate a notable difference in the antibacterial activity of the hydrogels against gram-positive (*S. aureus* and *E. faecalis*) versus gram-negative (*E. coli* and *P. aeruginosa*) bacteria, with a stronger effect seen against the gram-positive strains. This is likely due to differences in complicity of the cell wall structure between the gram-positive and gram-negative bacteria, since gram-positive bacteria have less complex cell wall structure compared to gram-negative bacteria [69,70].

In the next phase, both crosslinked and non-crosslinked PVA/PVP/HP-3 hydrogels were selected to be further investigated and characterized to assess the impact of the chemical and physical crosslinking (F/T cycles), since products containing 3% HP are the clinically approved concentration for medical applications and suitable for industrial use. Furthermore, its antibacterial activity was found to be similar to that of the higher 5% concentration, making the 3% concentration both practical and efficient, as it eliminates the need for a higher concentration, reduces production costs, and minimizes the use of excess chemicals, making the process more environmentally sustainable. Regarding the F/T cycled hydrogels, our results showed no significant differences between the hydrogels throughout the experiment (Figure 3C), with both 1 and 5 F/T cycle hydrogels, showing stronger effects against gram-positive bacteria. Collectively, these results demonstrate the long-term antibacterial effect of hydrogels, underscoring their potential use in medical applications such as wound-healing devices.

### 2.3. Controlled Release Kinetics of HP from the Hydrogels

**Figure 4:**
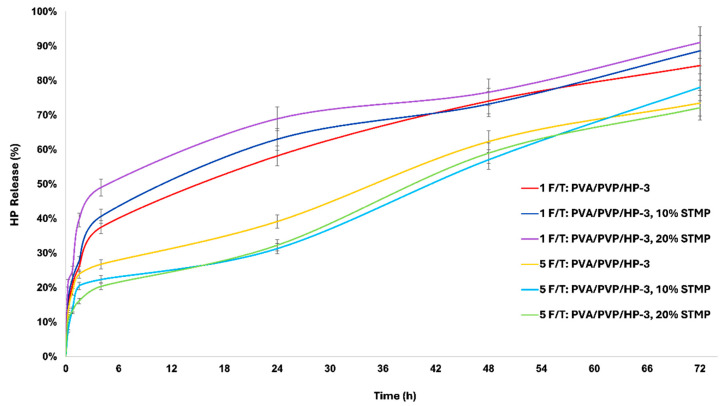
Kinetics of the HP controlled release rate (percentage) of the hydrogels subjected to 1 F/T: PVA/PVP/HP-3 (red), PVA/PVP/HP-3, 10% STMP (blue), and PVA/PVP/HP-3, 20% STMP (purple) and subjected to 5 F/T: PVA/PVP/HP-3 (yellow), PVA/PVP/HP-3, 10% STMP (light blue), and PVA/PVP/HP-3, 20% STMP (green).

Release behavior tests were conducted to characterize the release profiles of HP from the hydrogels. The kinetics of HP release were evaluated by placing the hydrogels on ex vivo skin model skin, as described in Section 4.2.4 and Section 4.2.5. Results presented in Figure 4 indicate a rapid release of HP within the first 4 h. For the hydrogels subjected to 1 freeze–thaw (F/T) cycle, nearly 50% of HP was released. Specifically, the PVA/PVP/HP-3 formulation released the lowest amount (37%), followed by PVA/PVP/HP-3, 10% STMP (40%), and PVA/PVP/HP-3, 20% STMP (49%). Conversely, hydrogels subjected to the 5 F/T cycles exhibited a slower release rate compared to the 1 F/T hydrogels, with PVA/PVP/HP-3 and the other two formulations showing similar release percentages (26%, 22%, and 20%, respectively).

A slow and moderated release was observed for all hydrogels after the initial 4 h period, continuing consistently over a 72 h period. After 72 h, the 1 F/T hydrogels reached a release percentage of 85–90%, whereas the 5 F/T hydrogels achieved a release of 72–78% from the loaded HP concentration.

These results suggest that chemical crosslinking did not significantly affect the controlled release behavior of the hydrogels. However, an increase in crosslinking in the 1 F/T hydrogels led to an increased HP release rate. This phenomenon can be attributed to the reduction in hydrogen bonds between the hydrogel chains and HP due to crosslinking occupancy. Conversely, the 5 F/T hydrogels were less influenced by chemical crosslinking compared to physical crosslinking (due to hydrogen bonds). The chemically crosslinked hydrogels exhibited a slightly more moderated HP release rate than the non-crosslinked ones, although the overall release rate of the 5 F/T hydrogels was lower. Physical crosslinking appears to strengthen the hydrogen bonds between the hydrogel chains and HP, making it more challenging for HP to be released from the hydrogels. Thus, chemical crosslinking has a less crucial impact on HP release behavior compared to physical crosslinking. It is important to note that these results do not directly reflect the outcomes of the in vitro experiments due to differing experimental conditions. However, they may explain why chemical crosslinking did not significantly affect antibacterial activity, as there was minimal variation in the HP release rate observed during the experiments.

### 2.4. Characterization

#### 2.4.1. Water-Swelling Capacity of the PVA/PVP Hydrogels

**Figure 5:**
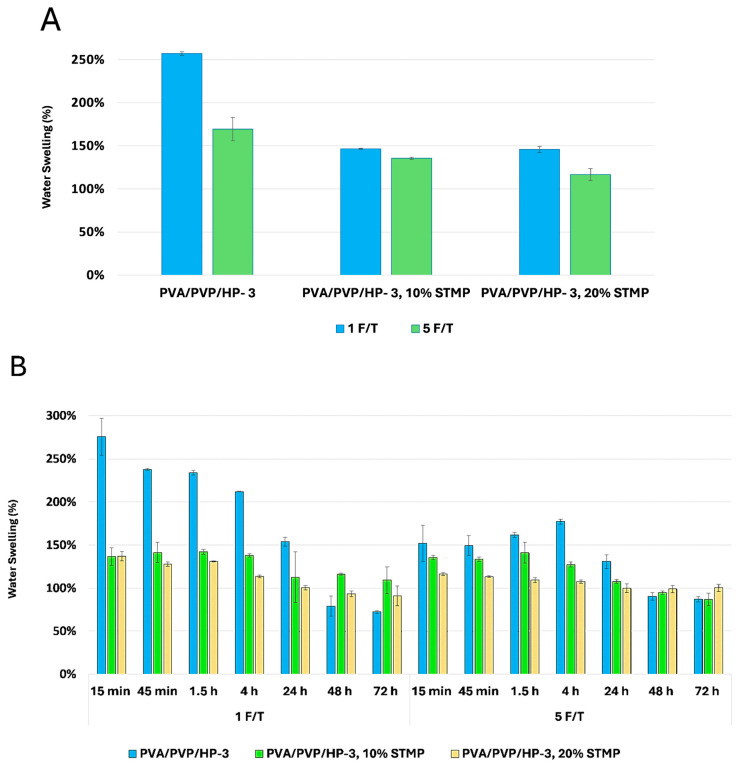
Water-swelling capacity of the PVA/PVP hydrogels after 24 h (**A**) and after applying onto the skin for 15 and 45 min, 1.5, 4, 24, 48 and 72 h (**B**): PVA/PVP/HP-3 (blue), PVA/PVP/HP-3, 10% STMP (green), and PVA/PVP/HP-3, 20% STMP (orange) treated by 1 and 5 F/T cycles.

The water-swelling content of deionized distilled water (DDW) in the PVA/PVP hydrogels was measured for 2 × 2 cm^2^ samples immersed in 40 mL DDW for 24 h. The swelling ratio was calculated using Equation (1), as described in Section 4.3.1. Figure 5 illustrates that all PVA/PVP hydrogels exhibited a swelling greater than 100% compared to their original weight. Specifically, Figure 5A shows that PVA/PVP/HP-3 with only 1 F/T exhibited water swelling of over 250%, whereas the same composition with 5 F/T exhibited water swelling of approximately 170%. Additionally, an increase in chemical crosslinking led to a slight reduction in swelling capacity, with 10% and 20% crosslinking resulting in swelling percentages of 146% and 143%, respectively. This reduction is due to the crosslinking of STMP with the PVA through phosphorylation [28], presenting an intramolecular covalent crosslinked structure. Consequently, water swelling decreased by almost 100% as shown in Figure 5A. The freeze–thaw (F/T) cycles notably impacted the hydrogel swelling capacity, as seen in Figure 5A, demonstrating an inverse relationship between the water-swelling ratio and the number of F/T cycles. As the number of F/T cycles increased, the swelling ratio decreased. This reduction in swelling can be attributed to the effect of F/T cycles, which increase the number and strength of hydrogen bonds between the PVA and PVP polymeric chains. Increased F/T cycles create a stronger three-dimensional hydrogel network [71], thereby reducing the penetration of water molecules. Figure 5B illustrates the water-swelling behavior of non-crosslinked and crosslinked hydrogels (PVA/PVP/HP-3, PVA/PVP/HP-3, 10% STM, and PVA/PVP/HP-3, 20% STMP) after being applied onto the skin model for various duration periods (15 and 45 min, 1.5, 4, 24, 48, and 72 h), followed by immersion of the hydrogel pieces in DDW, as described in Section 4.3.1. This procedure was performed to evaluate their swelling capacity for potential repeated use. During the first 24 h, all samples exhibited over 100% swelling. However, after 48 and 72 h, the non-crosslinked hydrogels showed a significant decrease in swelling capacity. Specifically, the 1 F/T hydrogels decreased to 78% and 72%, respectively, while the 5 F/T hydrogels decreased to 90% and 86%, respectively.

In contrast, the swelling capacity of the crosslinked samples remained relatively stable over time. For the 1 F/T hydrogels prepared with 10% crosslinker, the swelling percentages were 116% at 48 h and 109% at 72 h, compared to 112% at 24 h. The 5 F/T hydrogels prepared with 10% crosslinker showed swelling percentages of 94% at 48 h and 86% at 72 h, compared to 107% at 24 h. For the 1 F/T hydrogels with 20% crosslinking, the swelling percentages were 93% at 48 h and 91% at 72 h, compared to 100% at 24 h. The 5 F/T hydrogels prepared with 20% crosslinker exhibited swelling percentages of 98% at 48 h and 100% at 72 h, compared to 99% at 24 h. Overall, these results indicate that while non-crosslinked hydrogels significantly lose their swelling capacity over time, crosslinked hydrogels maintain a more consistent swelling behavior and are able to reach a similar original weight after swelling, making them potentially more suitable for repeated use.

#### 2.4.2. FTIR/ATR Spectra of the PVA/PVP Hydrogels

**Figure 6:**
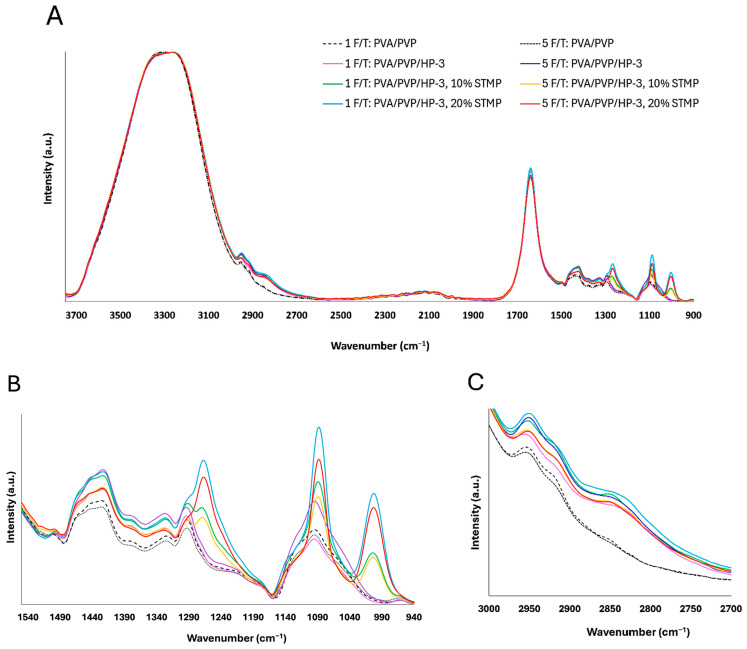
FTIR/ATR spectra of PVA/PVP (control), PVA/PVP/HP-3 and PVA/PVP/HP-3, 10%, and 20% STMP and treated with 1 or 5 F/T cycles (**A**), FTIR/ATR spectra in the range of 940–1550 cm^−1^ (**B**) and in the range of 2700–3000 cm^−1^ (**C**).

FTIR/ATR measurements were performed to identify functional groups in the different PVA/PVP hydrogels. The spectra consistently showed typical PVA bands, including a broad absorbance from 3100 to 3600 cm^−1^, which are related to the stretching of intermolecular and intramolecular hydrogen-bonded O–H groups [68]. Additionally, double bands within the 2950–2920 cm^−1^ range were observed, corresponding to the asymmetric and symmetric stretching of CH_2_ from alkyl groups [15] as illustrated in Figure 6. As expected, no significant differences were found between the hydrogel films, due to their uniform deacetylation degree (99+%) and consistent polymeric composition.

In contrast, the FTIR spectra of crosslinked hydrogels with STMP exhibited new characteristic peaks indicative of pyrophosphate systems [28]. Peaks at 1265, 1086, and 1001 cm^−1^ were identified as corresponding to O=P–O and C–O–P stretching and O–P–O bending, respectively [72,73,74]. These peaks confirmed the successful crosslinking of PVA chains with STMP [28,72]. Furthermore, the intensity of these wavenumbers increased with higher crosslinking ratios but diminished with an increased number of freeze–thaw cycles.

The shoulder observed at 2837 cm^−1^ is attributed to the unique O–H stretching vibration of bound HP as seen in Figure 6C [75]. The peak at 1498 cm^−1^ corresponds to the vibration of –CH_2_ groups, with its intensity increasing as hydrogel crystallinity grows, particularly under higher crosslinking ratios and multiple freeze–thaw cycles. The peak at 1093 cm^−1^ associated with C–O bending [30,76] becomes more pronounced with the addition of hydrogen peroxide due to increased internal bonding but shifts to a lower wavenumber (1088 cm^−1^) when STMP is introduced. Additionally, the prominent peak at 1648 cm^−1^ in the PVP control is typically linked to carbonyl groups [76]. However, it is more likely associated with C–N stretching at 1289 cm^−1^ in PVP [77] as shown in Figure 6B. This peak overlaps with the water-associated band [73], given that all samples contain 79% water.

#### 2.4.3. E-SEM Surface Morphology of Hydrogels

**Figure 7:**
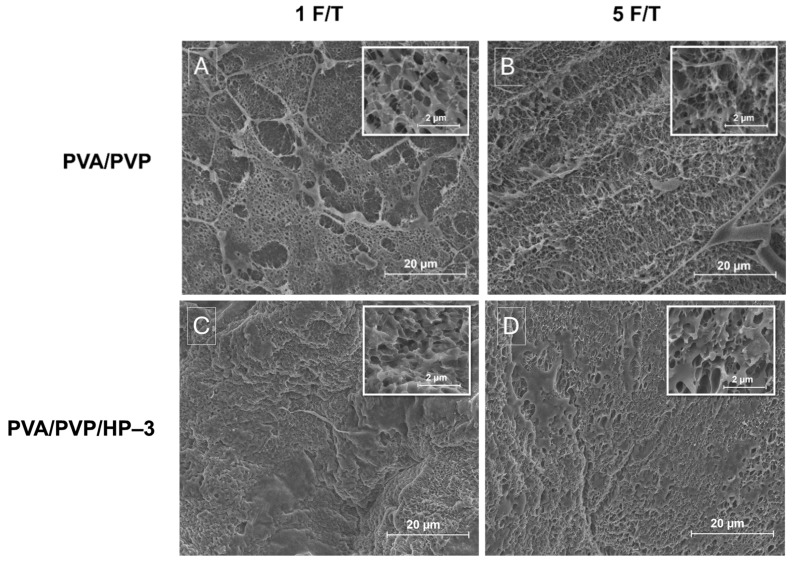

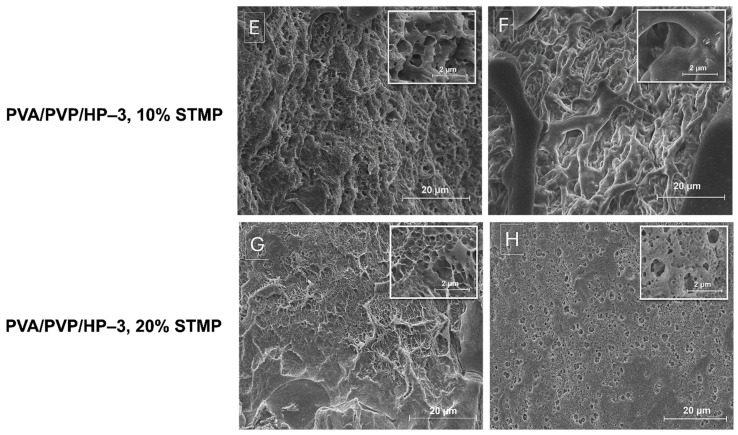
E-SEM surface images of the hydrogels treated with 1 or 5 F/T cycles: PVA/PVP (**A**,**B**, respectively), PVA/PVP/HP-3 (**C**,**D**, respectively), PVA/PVP/HP-3, 10% STMP (**E**,**F**, respectively), and PVA/PVP/HP-3, 20% STMP, respectively (**G**,**H**).

E-SEM surface images were used to investigate the effects of crosslinking process and freeze–thaw (F/T) cycles on the surface morphology of the various hydrogels. For the PVA/PVP hydrogel, E-SEM images showed that increasing the number of F/T cycles led to a more uniform and ordered structure in the 5-cycle samples compared to the 1-cycle samples (Figure 7A,B). Figure 7C,D demonstrate that in the PVA/PVP/HP-3 hydrogel, more F/T cycles resulted in a more porous structure. This increase in F/T cycles promotes the migration of PVA chains toward polymer-rich regions, promoting phase separation, crystallization, and gelation. These processes enhance mechanical strength, elasticity, water resistance, and hydrogel stability [29] due to the formation of locally ordered nanocrystalline domains [28] from initially random PVA and PVP chains during freezing. Upon thawing, the larger volume of ice crystals compared to water is maintained, leading to increased pore size and a more uniform structure [78,79].

Additionally, SEM images of the crosslinked hydrogels PVA/PVP/HP-3 prepared with 10% and 20% STMP in both F/T cycles (Figure 7E,H, respectively) revealed notable morphological differences. The 10% STMP-crosslinked hydrogel exhibits a mesh-like structure, whereas the 20% STMP variant shows a bulkier structure. The formation of agglomerates in these crosslinked samples is attributed to the phosphorylation mechanism and the incorporation of phosphate groups onto O–H groups [80]. As the STMP concentration increases, the hydrogel network becomes denser, resulting in a flatter, more wrinkled morphology with fewer surface pores.

## 3. Conclusions

PVA/PVP hydrogels encapsulated with hydrogen peroxide (HP) were developed and evaluated for their antibacterial activity, controlled release behavior, water-swelling capacity, and structural characteristics. The hydrogels were prepared with a constant PVA/PVP polymer ratio but varying HP concentrations (1.5%, 3%, and 5%). Modifications through freeze–thaw (F/T) cycles and chemical crosslinking with STMP were introduced to study their effects on HP release kinetics and the physical properties of the hydrogels.

Antibacterial activity assessed using a modified Kirby–Bauer disk diffusion assay against *S. aureus*, *E. faecalis*, *E. coli*, and *P. aeruginosa* revealed that all hydrogels exhibited antibacterial activity within 24 h, which increased over 72 h, particularly against gram-positive bacteria. Both crosslinked and non-crosslinked hydrogels demonstrated similar antibacterial performance, indicating that crosslinking did not significantly affect antibacterial efficacy. However, gram-negative bacteria (*E. coli* and *P. aeruginosa*) showed reduced antibacterial responses after 72 h, especially in hydrogels with lower HP concentrations.

Hydrogels with 3% HP were selected for further investigation as they provided effective antimicrobial activity comparable to the 5% concentration, while aligning with the clinically approved standards. Moreover, the controlled release process reduces the potential toxicity of HP. No significant differences in antibacterial activity were observed between hydrogels subjected to 1 and 5 F/T cycles, although stronger effects against gram-positive bacteria were seen across all samples.

HP controlled release kinetics revealed an initial rapid release during the first 4 h, followed by a slower, sustained release over the next 72 h. Hydrogels subjected to more F/T cycles exhibited slower HP release rates, while chemical crosslinking had minimal impact on release behavior. However, the inclusion of the chemical crosslinker enhanced the overall comfort and usability of the hydrogel, contributing to improved mechanical properties and handling. Additionally, non-crosslinked hydrogels demonstrated greater water-swelling capacity, which decreased with increasing F/T cycles and STMP concentration. Structural analysis via FTIR and E-SEM confirmed that crosslinking enhanced the crystallinity and structural integrity of the hydrogels. These results highlight that both physical and chemical modifications of PVA/PVP hydrogels significantly affect their controlled release, swelling behavior, and structural properties.

Therefore, based on the findings, it appears that the optimal formulation for ongoing work is the hydrogel with a minimal concentration of the crosslinker STMP (10%) and a 3% HP concentration (PVA/PVP/HP-3, 10% STMP). This is because chemical crosslinking had only a minor effect on release behavior, and the 3% HP concentration demonstrated antibacterial activity comparable to higher concentrations, making it an efficient and effective choice for future applications. Future work will focus on expanding the evaluation of these hydrogels through in vivo studies and potential clinical trials, with the goal of developing a practical wound-healing device for medical applications.

## 4. Materials and Methods

### 4.1. Materials

All materials were of analytical grade and purchased from commercial sources without further purification. Sodium hydroxide (NaOH), hydrochloric acid (HCl) 32%, PVA (99+% hydrolyzed average M.W 89,000–98,000), PVP K-30 (M.W 58,000), absolute anhydrous ethanol (EtOH, HPLC), sodium oxalate (Na_2_C_2_O_4_), potassium permanganate (KMnO_4_), and sulfuric acid 99.9%, etc., were purchased from Sigma Aldrich (Rehovot, Israel). Double distilled water (DDW) was obtained from a TREION purification system (Tel Aviv, Israel), and 30% hydrogen peroxide (HP) aqueous solution was purchased from Fisher Scientific Inc. (Pittsburgh, PA, USA).

### 4.2. Methods

#### 4.2.1. Preparation of PVA/PVP Hydrogels

**Figure 8:**
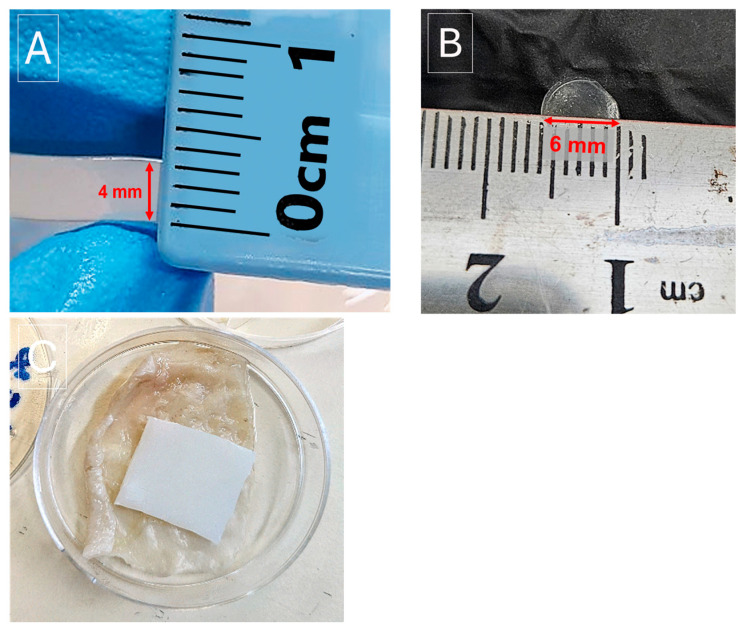
Digital images were captured to document the width of the formed hydrogels (**A**), the diameter of the hydrogel disk (**B**) and the square 2 × 2 cm^2^ dimension hydrogel piece applied on the dermis (inner side) of the skin in a Petri dish (**C**).

Hydrogels were prepared with different concentrations of HP (1.5%, 3%, and 5%), at the same polymeric composition ratio (3:1.2 *w/v*%) as described in Table 1. Briefly, 3 g of PVA was first completely dissolved in double distilled water (DDW) at 90 °C. After the solution cooled to 70 °C, 1.2 g of PVP was added and stirred until fully dissolved. Different concentrations of HP hydrogels were prepared by adding varying amounts of 30% HP solution to the mixture, as indicated in Table 1, to achieve the desired final concentrations, when cooled to 50 °C. Hydrogels with an average final thickness of 4 mm diameter (Figure 8A) were then produced by pouring the complete warm mixture into 100 × 10 mm^2^ Petri dishes. Air bubbles were removed as the solution cooled to room temperature, and then hydrogels were stabilized by freezing the hydrogels at −20 °C overnight. Hydrogels were thawed to room temperature before use. PVA/PVP hydrogels without HP were also prepared as control samples.

#### 4.2.2. Preparation of Chemically Crosslinked Hydrogel with STMP

PVA (3.0 g) was first dissolved in DDW at 90 °C, followed by adjusting the pH to 11–12 by adding 400 μL of 1M NaOH. The PVA crosslinker agent, STMP, was then added in different weight ratios to PVA (0.3 g and 0.6 g, 10 and 20% relative to the PVA content), and stirred for 30 min at 120 °C in a closed pressure suitable vail. The obtained aqueous solution was then cooled to 70 °C and acidified to pH 5 with 30 μL of 32%HCl. PVP (1.2 g) was then added until dissolution, followed by the addition of different volumes of 30% HP, as indicated in Table 2. Hydrogel with an average final thickness of 4 mm diameter (Figure 8A) was produced by pouring the complete mixture into 100 × 10 mm^2^ Petri dishes. Air bubbles were allowed to escape as the solution cooled to room temperature, and the hydrogels were stabilized by freezing at −20 °C. The hydrogels were thawed to room temperature before use. Crosslinked PVA/PVP without HP hydrogels were also prepared as control samples. Hydrogel compositions were prepared as mentioned in Table 2.

#### 4.2.3. In Vitro: Disk Diffusion Test to Assess the Anti-Septic Behavior of HP Controlled Release from PVA/PVP Hydrogels

An adapted disk diffusion assay was performed to assess antibacterial activity against 4 bacteria: *S. aureus* ATCC 29213, *E. faecalis* ATCC 29212, *E. coli* ATCC 25922, and *P. aeruginosa* (lab strain) (American Type Culture Collection (ATCC), Manassas, Virginia, USA). Eight types of 6 mm diameter hydrogel disks (Figure 8B) were examined: PVA/PVP, PVA/PVP/HP-1.5, PVA/PVP/HP-3, PVA/PVP/HP-5, crosslinked PVA/PVP/HP-1.5, 10% STMP, PVA/PVP/HP-3, 10% STMP, PVA/PVP/HP-5, 10% STMP, and PVA/PVP/HP-3, 20% STMP. Hydrogel disks were obtained by cutting the hydrogels using a custom-designed 6 mm inner diameter circular mold. For each experiment, on the first day, a bacterial stock was prepared by inoculating Luria–Bertani broth (LB, BD, Thermo Fisher Scientific, Manchester, UK) with a frozen bacterial stock and incubated overnight at 37 °C with continuous shaking at 250 RPM. The following day (day 2), the overnight stock was used to plate 8 LB-agar (BD, Difco, Thermo Fisher Scientific, Manchester, UK) plates (Petri dished with 8.5 cm inner diameter, Greiner, Rehovot, Israel) using a sterile swab. Each type of hydrogel disk was placed in the center of a swabbed plate forming the first set of plates to evaluate antibacterial activity over the initial 24 h. At the same time, two additional sets of 8 disks were placed in the center of 16 LB-agar plates (un-swabbed). The 24 plates were incubated at 37 °C (no shaking), together with a new bacterial stock (with shaking). The next day (day 3), the first swabbed set of plates was taken for inhibition zone diameter measurements. Another set of 8 plates was then swabbed with a fresh bacterial stock. The hydrogel disks, initially placed on the plates, were temporarily removed for swabbing and immediately returned to the same position. These plates served to test the antibacterial activity of the hydrogel on day 2 of the experiment. This set of plates, together with the last un-swabbed set of plates, were put back for incubation at 37 °C (no shaking). A new bacterial stock was also placed for incubation at 37 °C (with shaking). The following day (day 4), the second set of swabbed plates was taken for inhibition zone diameter measurement. The third set of plates was then swabbed with a fresh bacterial stock and incubated at 37 °C (no shaking). These plates served to test the antibacterial activity of the hydrogel on day 3 of the experiment. The next day (day 5), the last set of swabbed plates was taken for inhibition zone diameter measurement. 5 F/T-PVA/PVP, 5 F/T-PVA/PVP/HP-3, 5 F/T-PVA/PVP/HP-3, 10% STMP, and 5 F/T-PVA/PVP/HP-3, 20% STMP hydrogel disks’ antibacterial activity was similarly evaluated. At least 3 biological repeats were performed for each bacterial strain tested. Error bars represent standard deviation.

#### 4.2.4. Controlled Release Rate of HP from the PVA/PVP/HP Hydrogels on Ex Vivo Skin Model

The HP release rate from the HP-loaded PVA/PVP hydrogels was determined by cutting the prepared hydrogels into uniform square pieces with dimensions of 2 × 2 cm^2^. These pieces were then applied on top of the dermis (inner side) of chicken skin provided by a local butchery with approximately average dimensions of 4 × 4 mm^2^ in a closed 10 × 10 mm^2^ Petri dish (Figure 8C), for various time periods (0, 0.25, 0.85, 1.5, 4, 24, 48, 72 h) at 36 °C. After each time point, the hydrogel pieces were removed from the chicken skin and immersed in 40 mL of DDW overnight to extract the remaining HP content from the hydrogel slices. The concentration of HP released into the water at each time point was determined as described in Section 4.2.5 [81]. Release rate tests were performed in triplicate.

#### 4.2.5. Determination of HP Content of the PVA/PVP/HP Hydrogels

The HP content of the PVA/PVP/HP hydrogels was measured using a precalibrated (with sodium oxalate) 0.1M KMnO_4_ titer solution, according to a reported method [81]. Briefly, the hydrogel slices were incubated in 40 mL of DDW overnight to extract the HP content. After the kinetic experiments, the hydrogels were transferred to fresh DDW to further extract any remaining HP, accelerating the diffusion process. Following the immersion, the hydrogels were removed and 2.6 mL of 99.9% sulfuric acid was added to the solution and stirred before titration. The titrant was then pipetted into the solution while being magnetically stirred, until the solution color changed from transparent to purple, indicating the end of the titration. HP determination was performed in triplicate.

### 4.3. Characterization of the Hydrogels

#### 4.3.1. Swelling Test

The water swelling of the hydrogels was measured after the kinetic testing by immersing the hydrogels in a flask containing 40 mL of water for 24 h at 4 °C. The percentage swelling was calculated based on the changes in the weight of the swollen hydrogel slices compared to their original weight. This was done by recording the weights at desired time intervals after wiping off excess water from the surface with filter paper. The swelling percentage was calculated according to Equation (1):

(1) $$\%\mathrm{Swelling}=\frac{\left(W_{t}-W_{i}\right)\times 100}{W_{i}}$$

where W_i_ is the original weight and W_t_ is the weight of the swollen hydrogel after a designated time period.

#### 4.3.2. Fourier Transform Infrared (FTIR) Spectroscopy

Hydrogel compositions were characterized using the attenuated total reflectance (ATR) technique with a Bruker Alpha-FTIR Quick Snap™ sampling module equipped with a platinum ATR diamond module (Bruker, Billerica, MA, USA) and translated using the OPUS program version 7.0. The FTIR spectra were normalized, and major vibration bands were associated with chemical groups.

#### 4.3.3. Scanning Electron Microscope (E-SEM)

Surface morphology images were taken using a JEOL-JSM 840 SEM operating at 5 kV (Tokyo, Japan). Hydrogel samples were first lyophilized to extract all the water content and then attached with carbon tape, followed by iridium coating under a vacuum prior to imaging.

## Figures and Tables

**Table 1:** Composition of Materials Used for Preparation of Different Hydrogels (20 g total weight).

Sample Name	PVA (g)	PVP (g)	30% HP (ml)	DDW (g)
PVA/PVP	3	1.2	-	15.8
PVA/PVP/HP-1.5	3	1.2	1	14.8
PVA/PVP/HP-3	3	1.2	2	13.8
PVA/PVP/HP-5	3	1.2	3.3	12.5

**Table 2:** Composition of Materials Used for Preparation of Different Chemical Crosslinked Hydrogels (20 g total weight).

Sample Name	PVA (g)	PVP (g)	STMP (g)	30% HP (mL)	DDW (g)
PVA/PVP-10% STMP	3	1.2	0.3	-	15.5
PVA/PVP/HP-1.5, 10% STMP	3	1.2	0.3	1	14.5
PVA/PVP/HP-3, 10% STMP	3	1.2	0.3	2	13.5
PVA/PVP/HP-3, 20% STMP	3	1.2	0.6	2	13.2
PVA/PVP/HP-5, 10% STMP	3	1.2	0.3	3.3	12. 2

## Data Availability

The raw data supporting the conclusions of this article will be made available by the authors on request.
